# Lipids Composition in Plant Membranes

**DOI:** 10.1007/s12013-020-00947-w

**Published:** 2020-10-09

**Authors:** Emilia Reszczyńska, Agnieszka Hanaka

**Affiliations:** Department of Plant Physiology and Biophysics, Institute of Biological Sciences, Faculty of Biology and Biotechnology, Maria Curie-Sklodowska University, 20-033 Lublin, Poland

**Keywords:** Fatty acid, Lipid, Membrane, Plant

## Abstract

The paper focuses on the selected plant lipid issues. Classification, nomenclature, and abundance of fatty acids was discussed. Then, classification, composition, role, and organization of lipids were displayed. The involvement of lipids in xantophyll cycle and glycerolipids synthesis (as the most abundant of all lipid classes) were also discussed. Moreover, in order to better understand the biomembranes remodeling, the model (artificial) membranes, mimicking the naturally occurring membranes are employed and the survey on their composition and application in different kind of research was performed. High level of lipids remodeling in the plant membranes under different environmental conditions, e.g., nutrient deficiency, temperature stress, salinity or drought was proved. The key advantage of lipid research was the conclusion that lipids could serve as the markers of plant physiological condition and the detailed knowledge on lipids chemistry will allow to modify their composition for industrial needs.

Plants are constantly exposed to stress resulting from the conditions in which they are growing. They have to adapt to the external changes like humidity, salinity, or temperature. In order to maintain the normal physiological function and survive in the unfavorable environmental conditions, plants have developed defense mechanisms. Among them are alterations in the content of lipids, proteins or other molecules. For example, some of the plants are sensitive to temperature changes, e.g., *Cucumis sativa* L. [1] or *Solanum lycopersicum* L. [2], whereas others are less sensitive to temperature fluctuations, e.g., *Arabidopsis thaliana* L. [3] or *Spinacia oleraceae* L. These differences could be partially explained by the quantitative and qualitative changes in the lipid composition, which in turn triggers membrane fluidity and its function. Therefore, it is worth to present the selected lipid issues with the aim of explaining differences in their content, specific role in plants and emphasizing their impact in adverse conditions.

## Classification of Fatty Acids in Plants

Nowadays, structure and role of about 400 different fatty acids are known in the plant kingdom [4]. Some of them are inevitable in the proper function of plant cells and some have positive effects on human health (e.g., anti-inflammatory [5–7], anticancer [8, 9], antibacterial [10], and antiparasitic activity [11]) or are demanded in the different branches of industry, like food, pharmaceuticals, and cosmetics production [12–14].

The plant membranes are composed mainly of lipids which possess a hydrophilic, polar head attached to a glycerol backbone and a hydrophobic tail built of two fatty acids. Lipids form a hydrophobic barrier that separates cells and organelles from the environment [15, 16]. The core building block of fatty acids is a hydrocarbon chain with a carboxyl group (-COOH) located on its terminal end. Based on the chain length of fatty acids, they are classified as: short-chain (aliphatic tails of up to 5 or even 7 carbons), medium-chain (aliphatic tails of 6–8 up to 12–14 carbons), long-chain (aliphatic tails of 13–18 up to 22 carbons), or very long-chain fatty acids (aliphatic tails longer than 22 carbons; >C22) [17–21]. Most often, the number of carbon atoms in the plant tissues is between 14 and 24. Moreover, the aliphatic chain can be saturated (saturated fatty acid, SFA) or unsaturated (unsaturated fatty acid, UFA), where all carbon–carbon linkages form single bonds, or some carbons are matched by one or more double bonds, respectively. In addition, UFA can be divided into monounsaturated (monoenoic) fatty acids (MUFA) and polyunsaturated fatty acids (PUFA) with exactly one or at least two double bonds, respectively [22, 23]. Fatty acids are the building blocks of lipids.

## Nomenclature of Fatty Acids

**Fig. 1:**
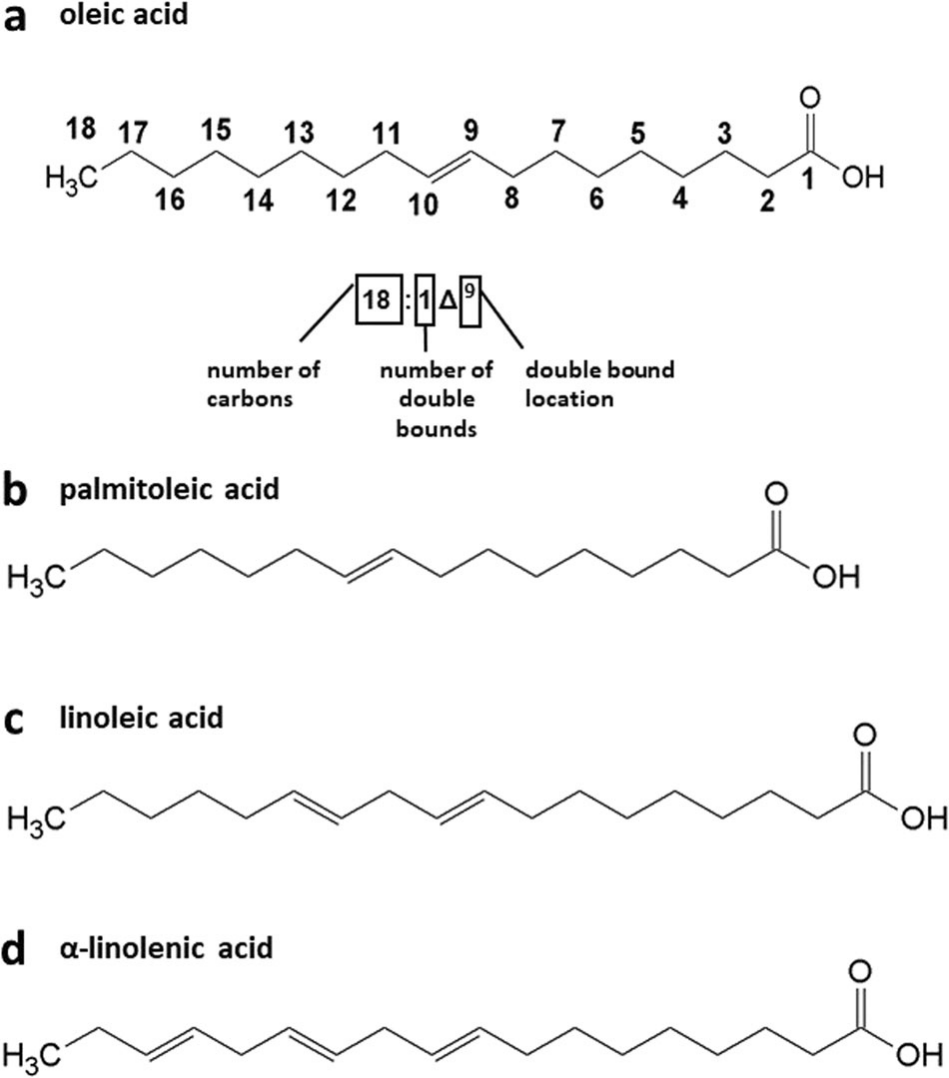
Exemplary formula of fatty acids. Fatty acids are numbered from -COOH group (Δ) and from -CH_3_ group (ω). **a** *Cis*-oleic acid—18:1—is with one double bound Δ^9^ (IUPAC: (9Z)-Octadec-9-enoic acid), **b** palmitoleic acid—16:1—is with one double bound Δ^9^ (IUPAC: (9Z)-Hexadec-9-enoic acid), **c** linoleic acid—18:2—is with two double bounds Δ^9,12^ (IUPAC: 9-*cis*,12-*cis* octadecadienoic acid), **d** α-linolenic acid—18:3—is with tree double bounds Δ^9,12,15^ (IUPAC: (9Z,12Z,15Z)-octadec-9,12,15-trienoic acid)

According to the International Union of Pure and Applied Chemistry (IUPAC) nomenclature of fatty acids, they can be formed using three systems of rules known as the shorthand formulas, the systematic names and the trivial names. The triple nomenclature can be demonstrated on one of the saturated fatty acids: C16:0—shorthand formula; hexadecenoic acid—systematic one; palmitic acid—trivial name. More complicated names can be constructed for the MUFA and PUFA, where one or more double bounds in acyl chain occur. In the case of the fatty acid possessing one double bound, C16:1 (n-7), it can be denoted as *cis*-hexadec-9-enoic acid (systematic formula) or palmitoleic acid (trivial name) [24]. For PUFA, two examples with different numbers of double bonds in the molecule are shown below in order to clarify the nomenclature. *α*-linolenic acid with the *cis* double bond located at the third region in carbon atom (n-3) marked from the end with methyl group is described as the omega (ω)-3 with the general structure CH_3_CH_2_(CH = CHCH_2_)_*n*_COOH, where *n* shows the numbering of *cis* double bond from the methyl terminus [24, 25]. In addition, the position of the double bond in the carbon chain can be designated by delta (Δ) before the full name of fatty acid, counting carbons from the carboxyl group [26, 27]. Linoleic acid (C18:2) with 18 carbon chain and two *cis* double bonds at C-9 and C12 from the carboxyl acid group could be specified as: 18:2 *cis*-Δ^9^, *cis*-Δ^12^ octadecadienoic acid; *cis, cis*-9,12-octadecadienoic acid or *cis,cis*-6,9-octadecadienoic acid. Sometimes PUFA are designated without ω (C18:3), but it is unequivocal and can be represented by a few different fatty acids: C18:3*ω*1, C18:3*ω*3, C18:3*ω*6, or C18:3*ω*9 [22, 23, 28]. The systematic names of fatty acids are derived from the names of the main straight chain by the substitution of suffix -e with -oic, e.g., hydrocarbon chain of C18 saturated fatty acid is octadecane and the acid is called octadecanoic acid (C18:0) [22]. The exemplary formula of fatty acid was presented in Fig. 1. Some trivial names of fatty acids origin from their natural sources, like palmitic acid, which was detected as a palm oil component; oleic acid (C18:1 *cis*-Δ^9^)—occurred in olive oil [24] and myristic acid (tetradecanoic acid)—was first identified in the *Myristicaceae* family [29].

## Fatty Acids Composition in Plants

Some of the plant families are more often implemented into the research on fatty acids. Among them are Fabaceae and Asteraceae. To the Fabaceae belong, i.e., *Arachis hypogaea* L. [30], *Astragalus* L. [31], *Pisum sativum* L. [32], and to Asteraceae: *Anthemis altissima* L. [33], *A. arvensis* L. [34], *A. talyschensis* L. [35], *Chamaemelum nobile* L. [36], *Tagetes patula* L. [37]. Other families also have their representatives in the experiments on lipids, e.g., Lauraceae with *Cinnamomum camphora* and *Umbellularia californica* [31], Vitaceae with *Cissus populnea* Guill. and Perr. [31], Polygonaceae with *Fagopyrum esculentum* and Brassicaceae with *Arabidopsis thaliana*. Species listed above are used in pharmacy and medicine, e.g., *Astragalus* (recommended in immune disorders) [31] and *A. altissima* (possess sedative, digestive and antimicrobial activity) [38–40]; nutrition, e.g., *A. hypogaea, F. esculentum* (applied in human diet); industry, e.g., *C. camphora* and *U. californica* (both used in biodiesel production, especially C12:0-14:0) [17]; and the research, e.g., *A. thaliana* [41].

The summary of various plant families, species and plant parts (such as the seeds, leaves, flowers, stem oils, and roots) in Table 1 shows the considerable quantitative and qualitative differences in the fatty acids composition. Below are presented some specific examples concerning the seeds, aerial parts, leaves, flowers, and leafy stems.

**Table 1:** The exemplary composition of fatty acid in the selected plant families

Fatty acids content [%]	*Asteraceae* *Anthemis* L.				*Fabaceae* *Pisum* L. [38, 143] *Arachis* L. [30]		*Brassicaceae* *Arabidopsis thaliana* L.	
	Flowers	Leaves	Seeds	Stem oil	Seeds	Leaves	Seeds	Roots
14:0	nd	nd	2.5^a^ [144]	nd	nd	nd	nd	nd
16:0	nd	nd	24.8^a^ [144]	39.6 [33]; 21.2 [42]	19.56 [38]; 14.96 [143]	15.0 [81];13.7 [135]	7.1 [34];10.2 [135]	26.8 [81];24.7 [135]
16:3	nd	nd	nd	nd	nd	13.8 [81];16.0 [135]	nd	nd
18:0 18:1 18:2 18:3	0.5 [35] 5.1 [35] 3.2 [35] nd	4.1 [35] 9.7 [35] 76.7 [35] nd	4.7^a^ [144] nd nd nd	nd nd 36.2 [33] nd	8.22 [38] 50 [30];11.37 [38];15.01 [143] 30 [30]; 38.94 [38]; 53.09 [143] 16.87 [38]; 9.52 [143]	nd 3.5 [81];12.3 [135] 15.7 [81];14.5 [135] 46.0 [81];50.8 [135]	4.3 [34] 13.4 [34];15.4 [135] 30 [30];26.3 [34];32.7 [135] 19 [30];16.2 [34];20.3 [135]	nd nd 35.4 [81], 29.8 [135] 30.8 [81], 29.1 [135]
20:0	nd	nd	nd	nd	2.64 [38]	nd	nd	nd
22:0	nd	nd	nd	nd	nd	nd	2.4 [34]	nd

*nd* no data

^a^Average value for the genus *Anthemis* calculated on the basis of five different species, i.e., *Anthemis cotula*, *A. macrotis*, *A. annua, A. austriaca,* and *A. santonicum* [144]

The same parts of the different plants can vary significantly in the composition of fatty acids, e.g., in seeds. The seeds of peanuts (*A. hypogaea*) contained the highest amounts of oleic (C18:1) and linoleic (C18:2) acids reaching 50% and 30%, respectively [30]. In the essential oil from the aerial parts of *A. arvensis*, the palmitic acid achieved ~21% [42], whereas 8.8% in the seeds with the total PUFA/SFA ratio equal 7.17 [34]. In the seeds of *C. populnea*, the most abundant among fatty acids were palmitic (C16:0)—40%, oleic (C18:1n-9)—27%, stearic (C18:0)—16.5%, and linoleic (C18:2n-6)—11.86% acids. Oil from the *C. papulnea* seeds contained SFA, which makes it appropriate for frying food because it is stable at increasing temperatures and stay resistant to oxidation [43]. The highest relative content of fatty acids in the *F. esculentum* seeds was determined for linoleic (C18:2n-6) (in the range 35.54–47.57%), oleic (C18:1n-9) (in the range 20.96–40.76%), and palmitic (C16:0) (in the range 13.86–26.42%) acids and the range of their values was dependent on the plant part (whole grain, hulls and bran). In addition, other fatty acids were identified in smaller quantities, i.e., lauric (C12:0), myristic (C14:0), palmitoleic (C16:1), stearic (C18:0), α-linolenic (C18:3n-3), and arachidic (C20:0) acids [44]. Moreover, α-linoleic acid is a precursor of the phytohormone, jasmonic acid, which is involved in the response of plants to the biotic and abiotic stress conditions [30]. Furthermore, both in the transgenic and non-transgenic seeds of *A. thaliana* the most abundant fatty acids were 18:2 (~30%) and 18:3 (~19%) [30] and the PUFA/SFA ratio was 4.05 [34].

The leaves of plants like *C. camphora* and *U. californica* in the presence of thioesterases accumulated 52 and 40% of C12:0 and C14:0, respectively, which protected plants against the fatty acids modification and deprivation of the membrane homeostasis. Triacylglycerols compose the fatty acids, e.g., C12:0, C14:0, C16:0, but their proportion depends on the expression or co-expression of thioesterases in the plants. Fatty acids are very important during modification of the lipid profiles in the plant membranes because their unbalance causes undesirable chlorosis and cell death [17]. In *A. talyschensis*, the composition of fatty acids depended on the plant part, thus SFA in the flowers was 1.3% and in the leaves—9.4% and UFA was 17.7% in the flowers and 87.0% in the leaves—being not detected in the stem. The proportion of PUFA/SFA in the flowers and leaves was 13.62 and 9.25, respectively [35]. In addition, both leafy stems and flowers of *C. nobile* contained fatty acids: C16:0 (~18%), C18:1n-9 (~23%), C18:2n-6 (~29%), C18:3n-3 (~18%), and the proportion of PUFA/SFA was 1.72 [36].

## Classification and Composition of Lipids in Plants

**Fig. 2:**
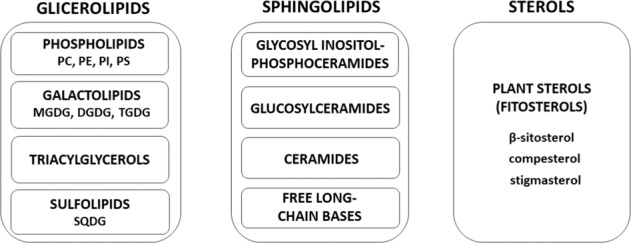
Classification of plant membrane lipids [142]

In the plant membranes, three main classes of lipids appear, i.e., glycerolipids, sphingolipids, and sterols (Fig. 2). The most abundant are glycerolipids, which are divided into four groups: phospholipids (PL), galactolipids (GL), triacylglycerols (TAG), and sulfolipids (SL) [45, 46]. Phospholipids containing phosphorus are major constituents of the membranes and they have different head groups modified by choline, ethanolamine, serine, or inositol and are described as phosphatidylcholine (PC), phosphatidylethanolamine (PE), phosphatidylserine (PS), and phosphatidylinositol (PI), respectively. Phospholipids are also characterized by different length and the degree of unsaturation of their fatty acyl chains. Variations in their properties have an impact on the membrane characteristics. This class of lipids is unevenly distributed between the different membranes in the cell [24, 47–49]. By contrast, in photosynthetic membranes of plants the major constituents are the nonphosphorus galactolipids divided mainly into two classes, monogalactosyldiacylglycerol (MGDG) and digalactosyldiacylglycerol (DGDG). Moreover, the nonphosphorous are also sulfolipids with sulfur-containing lipid, sulfoquinovosyldiacylglycerol (SQDG) [50]. Both MGDG and DGDG and SQDG are synthesized exclusively in the chloroplasts [51–54]. Of the grana thylakoid membrane area, 20–30% is occupied by lipids, and the most part by proteins or photosynthetic protein complexes [55, 56]. The thylakoid membranes in higher plants contain four glycerolipids: MGDG, DGDG, SQDG, and phosphatidylglycerol (PG) [57]. Of all chloroplast lipids, MGDG and DGDG can reach 52% and 26%, respectively [58, 59]. The exemplary composition of glycerolipids in the membranes of spinach chloroplasts and their thylakoids are presented in Table 2.

**Table 2:** Composition of lipids in the membranes of spinach chloroplasts and their thylakoids

Composition of lipids	MGDG	DGDG	PC	PG	SL
Outer membrane of chloroplast	17	29	32	10	6
Inner membrane of chloroplast	49	30	6	8	5
Thylakoids	52	26	4.5	9.5	6.5

The proportion of the lipids was calculated as the weight of the percentage of fatty acids [59]

Plant sphingolipids are grouped into four classes: glycosyl inositolphosphoceramides (GIPC), glucosylceramides (GCer), ceramides (Cer), and free long-chain bases (LCB) [60, 61]. They are built of a ceramide backbone composed of a long-chain base and a long-chain fatty acid matched by esterification. Both Cer and LCB can be phosphorylated and de-phosphorylated and Cer, additionally, glucosylated [62]. The composition of LCBs is mainly formed from phytosphingosine and its desaturated form, but others are also known, e.g., sphinganine and sphingenine [63]. The quantity of sphingolipids differs significantly depending on the plant species and tissues, but mostly reaches up to 10% of the total lipids in plants [64]. In the total amount of sphingolipids in the leaves of Arabidopsis, the ratios of GIPC:GCer:Cer:LCB were as follows 64:34:2:0.5%, proving that GIPC and GCer were the most prevailing [65]. In the tonoplast, sphingolipids were detected in the range from 10 to 20% of the total membrane lipids [66, 67].

In plants, over 250 different sterols (phytosterols) have been identified. Among them most frequently are detected these belonging to 4-desmethylsterols, i.e., campesterol, stigmasterol, and sitosterol [68, 69]. Phytosterols can appear in the forms of free sterols, steryl esters, steryl glycosides, and acylated sterol glycosides [69].

## Lipids Organization in Membranes

Fatty acids composition (with the proportion of saturated and unsaturated fatty acids) influences lipid composition (specific proportions) and organization in plant membranes. For example, the percentage content of lipids in the thylakoid membranes of green plants is as follows: MGDG~50%, DGDG~25–30%, SQDG~5–15%, PG~5–15% [70, 71]. The most popular fatty acids in the skeleton of plant galactolipids are 18:3/16:3 as 34:6 MGDG, 18:3/18:3 as 36:6 MGDG, 18:3/16:0 as 34:3 DGDG, and 18:3/18:3 DGDG in the approximate proportion: 80%, 16%, 16%, 70%, respectively [72]. The biological membranes have different composition and contain the domains in their structure, called rafts, which are enriched in sphingolipids and sterols with reduced level of unsaturated fatty acids, esp. in phospholipids [73]. It means that rafts are structures of lesser fluidity than non-raft areas.

Lipids perform many functions (Table 3). Among others, they influence performance, regulation, and physical properties of the membranes [74, 75], serve in the distribution, organization, and functioning of bilayer spanning proteins [76], are involved in compartmentalization of cells and organells and are integral components of the photosynthetic protein complexes of the electron transport chain [55]. Lipids can also form other structures, e.g., plastoglobules and stromules in the chloroplasts. Plastoglobules are lipid droplets enclosed in lipid monolayer, which is connected to the stroma leaflet of the thylakoid membrane. They can be found in high number in etioplasts and in plastids of senescent leaves. Stromules are tubular extensions of both chloroplast envelopes into the cytosol and filled with stroma, but deprived of thylakoids. The number of plastoglobules and stromules increases during environmental stresses [77, 78].

**Table 3:** Lipids role in plants and their importance for humans [27, 40, 145, 146]

Role and importance of lipids	
Plants	Humans
The main structural components of biological membranes	Nutrients (improve the quantity and quality of oils for food and feed)
Provide fluidity and flexibility in the membranes	Medical/pharmaceutical application in health disorders
Serve as permeable and selective barriers to the external environment of cells (membrane trafficking)	Cosmetics (storage oils that accumulate in seeds used, e.g., soaps and cosmetics)
Modulate the physical properties of membranes (their surface charges, curvature, or clustering of proteins)	Chemicals (storage oils used e.g., in paints and detergents)
Provide the integrity of cells and organelles (a hydrophobic barrier for the membrane)	Petrochemical industry (storage oils used as renewables for the production of biodiesel)
Key components in the establishment of organelle identity and dynamic	
Components of enzyme system (e.g., xanthophyll cycle)	
Mediators of interactions with numerous membrane-associated proteins (e.g., photosynthetic proteins)	
Signal molecules regulating cell metabolism	
Major regulators of many fundamental cellular processes (cell division, cell growth, and gene expression)	
Energy storage compounds	

Lipids composition undergoes remodeling in the face of various physiological [30, 73, 79–82], and environmental conditions [83–85]. Moreover, the artificial membranes are composed and applied to broaden our understanding of nature. The model membranes mimicking the natural ones are dedicated to determining the network organization and reorganization of the molecules, structural and functional interactions and mechanisms in a simplified composition combined of a few different lipids (mostly two to five) (Table 4). Models of the artificially formed membranes are involved in the research on the molecular membrane architecture and structure [59, 86–88] based on the fluorescence [88] and microscopic techniques [87], including photosynthetic performance [89], xanthophyll cycle analysis [87, 90, 91], and free radicals connection with the environmental stress in plants [92]. For example, the mixture of two lipids, MGDG:DGDG in 2:1 ratio can be applied as the model of plant lipids in thylakoids for the LHCII (light-harvesting complex) measurements [86].

**Table 4:** Proportion of lipids in the model membranes

Components proportion	References
MGDG:DGDG 2:1	[59, 86, 87]
MGDG:DGDG 1:2	[88]
MGDG:DGDG 30:70	[87, 91]
PC:MGDG 30.1:12.9	[90]
MGDG:DGDG:SQDG:PG 50:28:9:13 47:27:12:14	[89]
MGDG:DGDG:SQDG:EPG 40:30:15:15	[147]
PC:PE:PI:PG:PA 44:22:18:11:6	[92]
DGDG:MGDG:SL 73:24:2	[87]
POPG:DGDG 1:1	[88]

*MGDG* monogalactosyldiacylglycerol, *DGDG* digalactosyldiacylglycerol, *PC* phosphatidylcholine, *SQDG* sulfoquinovosyldiacylglycerol, *POPG* 1-palmitoyl-2-oleoyl-*sn*-glycero-3-phosphocholine, *EPG* Egg phosphatidylglycerol; *PA* phosphatidic acid, *SL* sulfoquinovosyldiglyceride, *PI* phosphatidylinositol, *PE* phosphatidylethanolamine, *PG* phosphatidylglycerol

**Fig. 3:**
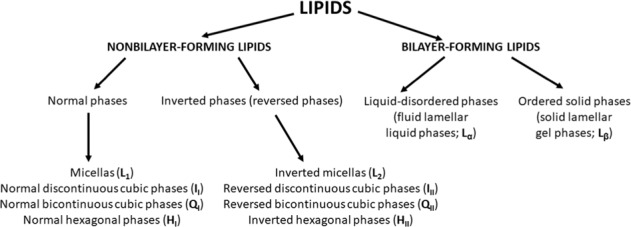
Division of lipids based on the type of the lipid phase produced in aqueous systems

Based on the type of the lipid phase produced by lipids in the aqueous systems, we differentiate the nonbilayer- and bilayer-forming lipids. Nonbilayer-forming lipids form the ordered solid phases and bilayer-forming ones—liquid-disordered phases [93] (Fig. 3). The nonbilayer-forming lipids possessing small polar head groups like MGDG and PE with elevated content of PUFAs form inverted micelles or tubular structures due to their cone-like shape and form an inverted hexagonal (H_II_) phase when dispersed in the aqueous solutions. The important functions of MGDG are to promote membrane stacking, stabilizing the inner membrane leaflet in grana disc [88] and conservation of photosynthetic energy [94]. Furthermore, the proportion of the thylakoid nonbilayer lipids are crucial, because the higher content of the MGDG is responsible for the membrane permeability and thermal stability of PSII [71].

The bilayer-forming lipids with large head groups, such as DGDG, SQDG, PC, PG, and the decreased content of long-chain PUFAs exhibit a cylindrical shape and form the lamellar L*α* phase [72, 93–96]. However, the increased ratio of DGDG to MGDG enhanced the stability of the thylakoid membrane [72]. Protein arrays are related with the phase transition of MGDG from a bilayer to a nonbilayer H_II_ phase which was observed in the stress conditions, e.g., cold, low light [97], osmotic stress, and in fatty-acid mutants [98]. An association between lipids and protein organization is explained by the lateral membrane pressure hypothesis [99] known as ‘force from lipids’ (FFL) principle [100].

## Lipids in Xanthophyll Cycle

The plants have developed the unique photoprotection mechanism, which prevents the excess absorption of light energy and consequently protects the photosynthetic apparatus from the oxidative damage. This process is called xanthophyll cycle [101]. In the xanthophyll cycle, the conversion of violaxanthin into zeaxanthin is done by violaxanthin deepoxidase (VDE) under high light [102–104]. VDE localizes to the thylakoid lumen and is regulated by lumen pH [90, 105] and by binding to MGDG. It means that the MGDG molecules can serve as the docking sites for the xanthophyll cycle enzymes. In chloroplasts, H_II_ can be established by MGDG, but in vitro VDE can also be stimulated by binding to PE [106]. The xanthophyll cycle pigments are located in the hydrophobic region of membrane with an easy access to the H_II_ phase [93, 107].

The studies concerning the location of the xanthophyll cycle in the transient membrane domain combined with LHCII, MGDG, VDE allowed to prove that MGDG have a crucial function in the stabilization of the structure of the LHCII protein in prevention its aggregation in PSII [71].

## Synthesis of Glycerolipids in Plants

In plants, the most abundant class of lipids are glycerolipids, therefore first, their synthesis based on two pathways, then a brief view of the synthesis of PL, GL, TAG, and SL are presented.

**Fig. 4:**
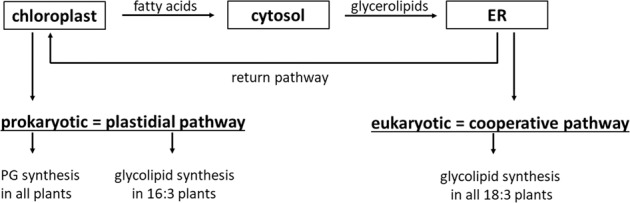
Prokaryotic and eukaryotic pathways [45, 110, 113]

Fatty acids are incorporated into glycerolipids in two different ways called the prokaryotic (plastidial) and the eukaryotic (cooperative) pathways located in the chloroplast and ER, respectively (Fig. 4) [15, 16, 108, 109]. The prokaryotic pathway is involved in PG synthesis in all plants, but in the glycerolipid synthesis only in 16:3 plants (which means 16 acyl carbons and 3 double bonds) in the *sn*-2 position of MGDG molecule [110]. Moreover, 16:3 plants are those which produce up to half of the MGDG in the plastidial pathway [111]. The eukaryotic pathway is involved in the glycerolipid synthesis in all plants, but mostly in 18:3 plants (which means 18 acyl carbons and 3 double bonds) in the *sn*-2 position of MGDG molecule [110]. Irregardless pathway type, biosynthesis of membrane lipids starts from the formation of PAs, which are utilized to produce plastidic lipids or phospholipids. Phosphatidic acid produced in the chloroplasts can be converted to diacylglycerol (DAG), which then serves as a precursor for plastidic lipid synthesis [45, 110, 112, 113]. PA is an intermediate molecule in the lipid synthesis and can be converted to and from PC and DAG because of the low energy requirements to remove them from membranes. PC could be a substrate for MGDG synthesis and DAG can be synthesized *de novo* with fatty acids, then removed from other lipids or derived from TAG turnover [111].

The prokaryotic pathway synthesizes four classes of glycerolipids: three glycolipid classes, i.e., MGDG, DGDG, trigalactosyldiacylglycerol (TGDG), and one sulfolipid class, SQDG. In the plant cell, 95% of fatty acids are produced by the plastidial fatty-acid synthase (FAS) belonging to the type I FAS [114]. First, coenzyme A (CoA) is converted into malonyl-CoA by acetyl-CoA carboxylase (ACCase) dependent on light [15]. Next, malonate is transferred to acyl carrier protein (ACP) by malonyl-CoA:ACP malonyltransferase (MCMT). Then, the activity of 3-ketoacyl-ACP synthase 3, 1, and 2 (KAS III, I, and II, respectively) give the main products of fatty-acid *de novo* synthesis: 16:0-ACP and 18:1Δ9-*cis*-ACP. Fatty-acid desaturation continues at high rate in the dark period [82].

By contrast, the eukaryotic pathway produces six phospholipid classes, i.e., phosphatidic acid (PA), PC, PE, PG, PI, and PS [115]. The eukaryotic pathway of 16:0/18:0 DAG moieties can produce around 20% of total DGDG. During the life cycle of plants, an active lipid exchange between the chloroplast and ER occurs *via* the import of the DAG moiety of PCs from the ER to the chloroplast envelope where it contributes to the DAG pool used to synthesize plastidic lipids [45, 110, 112, 113].

Phosphatidylgycerol is synthesized in plastids, ER, and mitochondria and in chloroplasts it is predominantly synthesized *via* the prokaryotic pathway. Phosphatidylcholine, PE, and PI are synthesized in the ER membrane. Triacylglycerol is mainly synthesized in the ER and chloroplastic envelope membranes and accumulates within the membrane bilayer and subsequently forms lipid droplets in the cytosol [116, 117].

Moreover, some plant species show various proportions between pro- and eukaryotic pathways [15]. For example, in Arabidopsis leaves in controlled conditions, ~50% of the chloroplastic glycolipids (MGDG, DGDG, and SQDG) are derived from the eukaryotic pathway, in which glycerolipids synthesized in the ER membrane are transferred to chloroplasts and converted into glycolipids [16, 118–120]. Other plants produce only one lipid, PG, in the prokaryotic pathway [15] and as the result of evolution [121], this pathway diminished in 18:3 plants [15]. Due to the insufficient information on prokaryotic pathway in 16:3 plants [110], the research of this pathway will provide data allowing to better understand the physiological significance of the lipid evolution in plants.

The phospholipid biosynthesis can be divided into the assembly of the phosphatidic acid (PA), formation free or activated DAG, which may be the sources for the biosynthesis of the cellular glycerolipids [122], and formation of the head group to form the whole glycerolipid molecule [47]. Both, PE and PC are synthesized in plants in two main steps. The first one is the conversion of serine to ethanolamine catalyzed by serine decarboxylase and the next one is the attachment of phosphocholine or phosphoethanolamine to the DAG backbone, catalyzed by aminoalcohol aminophosphotransferase [47]. Free fatty acids are exported from chloroplasts [123, 124].

The first step of galactolipid synthesis is the transfer of galactose from uridine diphosphate (UDP)-galactose (UDP-Gal) onto DAG in the presence of MGDG synthases. The second step is the transfer a galactose from UDP-Gal onto MGDG accompanied by digalactosyldiacylglycerol synthases. Both are localized to the outer envelope. Moreover, in order to introduce double bonds in MGDG and DGDG, different plastidial desaturases are synthesized in the inner [52, 53, 115, 125, 126] and outer envelope [114].

Synthesis of TAGs can be driven by different pathways. The most straightforward seems to be the pathway in which the acyltransferases were required for successive acylation of medium-chain fatty acid in the *sn*-2 and *sn*-3 position of TAGs. Then, diacylglycerol acyltransferase (DGAT) incorporated, e.g., PC molecules, onto the membrane [17, 19, 127–130]. TAGs can also be synthesized by PC involvement by application of its entire DAG molecule or acyl-CoA may be used as an acyl donor [15]. TAG is formed from the conversion of the DAG and in reaction of acylation, DAG can be converted to TAG [108].

Biosynthesis of SQDG comprises three enzymatic steps. Uridine triphosphate (UTP) and glucose-1-phosphate under action of the unique stroma-localized UDP-Glc pyrophosphorylase UGP3 produced UDP-glucose (UDP-Glc) [131]. Then, UDP-Glc and sulfite are converted into UDP-sulfoquinovose in the presence of stroma-localized UDP-sulfoquinovose synthase (SQD1) [132]. Next, sulfoquinovose is transferred to DAG and catalyzed by SQD2 localized in the inner envelope [99].

Understanding of lipids metabolism is essential to study their regulatory role in the plant growth and development.

## Fatty Acids and Lipids Composition under Adverse Conditions

Fatty acids and lipids are examined in the research on the reconstitution of membrane system and the effects of stress conditions. The disturbed balance of the membrane caused reorganization of the lipid bilayer [126]. The influence of adverse environment, e.g., nutrient deficiency (especially nitrogen and phosphate), temperature stress (heat, cold, and freezing), salinity and drought on membrane lipid composition was expansively proved (Table 5). Fatty acids and lipids composition were dependent on the length of time incubation in adverse conditions [41] or the level of the unfavorable agents [133]. The observed trends can vary from an increase in the total amount of each lipid class [134] to the elevation [85, 135] or decrease [136] of the specific fatty acids.

**Table 5:** Lipid composition in plant membranes under adverse environmental conditions

Plant species and environmental condition	Membrane remodeling	Implication	References
*A. thaliana* Nutrient stress— nitrogen deficiency	↓ MGDG (16:3/18:3) **↑** DGDG (16:0/18:3) **↑** phospholipids **↑** 12:0, 14:0, 16:3 No change in PE or PC, PS	N starvation did not result in the replacement of N containing glycerolipids with glycolipids; low N content in glycerolipids might explain why N deprivation did not affect the amounts of PC, PE and PS, but rather resulted in the remobilization of N from protein-bound amino acids	[148]
*A. thaliana* Nutrient stress— phosphate deficiency	↓ PC, PE, and PG ↑ DGDG and SQDG ↓ 18:0, 18:1 ↑ 18:2, 18:3	The replacement of phospholipids (PC, PE, and PG) with phosphorus-free glycolipids (DGDG and SQDG); phosphate-saving mechanisms include conversion of phospholipids into glycolipids, mainly DGDG	[51, 132, 149, 150]
*A. thaliana Pisum sativum* L. Temperature stress—heat (30–45 °C)	↑[136, 151]/↓[85]16:0, 16:3, 18:1, 18:2 **↓** MGDG, DGMG, SL, SQDG, PE, PG **↓** MGDG/DGDG **↓** 18:3-PG; 16:3-PG **↑**[136][151]/**↓**[135]18:3 **↑** 16:1	A chloroplast heat-inducible lipase (HIL1) stimulates DGDG synthesis and hydrolyzation of 18:3 from MGDG as a turnover of 18:3 under heat stress, where the liberated 18:3-FFA seems to be partly converted to TAG; the induction of genes encoding enzymes for galactolipid and sulfolipid synthesis and degradation of phospholipids; TAGs incorporated products derived from lipid metabolism such as DAGs and fatty acids (*via* PCs), thereby protecting the photosynthetic apparatus and increasing thermotolerance	[17, 85, 134–136, 151]
*A. thaliana* (leaf, seed, root) *Triticum aestivum* L*.* (leaves) *Nicotiana tabacum* Maize—leaf Temperature stress—cold (1–8 °C)	**↑** 16:3 (Arabidopsis leaves), 18:3 (*A. thaliana* roots and *N. tabacum* leaves) **↑** PA, DGDG (maize leaves) **↓** PC, MGDG (maize leaves) **↓** phospholipids containing saturated acyl groups ↑ MGDG ↓ DGDG ↑ PC, PE (*N. tabacum*, *A. thaliana*)	Enhanced turnover of PC to PA, which serves as precursors for galactolipid synthesis under low temperature conditions; 18:3 maintained membrane fluidity at low temperatures for plant survival under chilling conditions; TAGs are synthesized from MGDG after freezing induction	[84, 85, 134, 135, 151, 152]
*Triticum aestivum* L. *A. thaliana* (leaves) Temperature stress—freezing ((−2)–(−8) °C)	↑ PA ↓ PC, PE, PG ↓16:0 (*T. aestivum*)	The large decline in major membrane phospholipids but not galactolipids suggested that phospholipases were activated to a greater extent than galactolipases	[152]
*Aster tripolium* L. Salinity (200–855 mM NaCl)	↓ 16:1-*trans* ↑ 16:0, 18:0 ↓ 18:3 ↑ 18:2 ↑ glycolipids ↓ PC, PE	Salinity led to ↑ SFA, ↓ PUFA, and ↓ 18:3 ↑ 18:2 relative concentrations, which was expressed as a reduction of the fluidity of the chloroplast membrane and such membrane remodeling was connected with the adaptation to saline environment and protection against the oxidative effects of salt ions; 16:0 was the part of PSII protecting it during the accumulation in PSII; 18:2 was nonenzymatic ROS scavenger and cellular ROS controller	[137, 153]
*Brassica napus* L. (seeds) Drought	↓ 18:1 [154]↓/↑[138] 18:2 ↑ 18:3 ↓/↑[138] 16:0 [138]↓/↑ 18:0 ↓/↑[138]16:1 ↓ PG, PS, DGDG, SQDG	The delivery of stearic or oleic acid was limited by the stress; no effects were observed for eicosenic or erucic acid; less unsaturated species from several polar glycerolipid classes accumulated (PG, PS, DGDG and SQDG); MGDG acyl chains may be utilized for biosynthesis of SQDG; overexpression of two desaturation enzymes (FAD3 and FAD8) resulted in ↑ in linolenic acid and enhanced drought tolerance indicating defense mechanisms	[138, 154]

*MGDG* monogalactosyldiacylglycerol, *DGDG* digalactosyldiacylglycerol, *PC* phosphatidylcholine, *SQDG* sulfoquinovosyldiacylglycerol, *PA* phosphatidic acid, *SL* sulfolipid, *PI* phosphatidylinositol, *PE* phosphatidylethanolamine, *PG* phosphatidylglycerol, *PS* phosphatidylserine, *TAG* triacylglycerol, *DAG* diacylglycerol

12:0—lauric acid, 14:0—myristic acid, 18:0—stearic acid, 18:1—oleic acid, 18:2—linoleic acid, 18:3—linolenic acid, 18:3-PG— linolenic acid containing phosphatidylglycerol, 16:0—palmitic acid, 16:1—palmitoleic acid, 16:1*-t*—*trans*-Δ^3^-hexadecenoic acid, 16:3—hexadecenoic acid

*FT protein-PC flowering locus T (FT)* protein binding to phosphatidylcholine, *FAD3* fatty-acid desaturase 3 (cytosolic), *FAD8* fatty-acid desaturase 8 (plastidic)

There is a direct linkage between the variations in membrane fluidity and the changes in membrane thickness [137]. The content of UFA in lipid membranes increases with decreasing temperature, but constitutively higher levels of UFA do not lead to drought tolerance [138]. Moreover, higher quantity of PUFA in the seeds may result in their earlier maturity [138]. In the membranes, lipids are required for photosynthetic thermostability during elevation of temperature [139, 140]. The right temperature is necessary to protect and stabilize the photosystems, allowing the plant to maintain a functional and efficient photosynthetic machinery [141]. Temperature elevation reduces the membrane thickness by hydrophobic interaction in the membrane [76]. Oppositely, upon lowering the environmental temperature lipid bilayers become more ordered and as a consequence they become thicker [137].

On the basis of Table 5, it seems that lipids may serve as the biomarkers susceptible for various environmental stresses. Therefore, our understanding of plant lipid biosynthesis and chemistry is essential for manipulation in lipids *via* biotechnology and implementing the results in different industrial sectors beneficial for humans, e.g., pharmacy, cosmetics, chemistry, nutrition (Table 2), e.g., Arabidopsis genes can be employed for decreasing the undesirable fatty acids in *Nicotana tabacum* [134].

## Conclusions

The biosynthesis and lipid composition (the ratio of saturated to unsaturated acids) of biomembranes play a key role in the functioning of plants. During their growth, plants adapted to the adverse conditions through the reorganization of lipid membranes resulting from the change in the fatty-acid content and, consequently, the formation of lipids.

High level of lipids remodeling in plant membranes under different adverse conditions (e.g., nutrient deficiency, temperature stress, salinity, or drought) was proved. The elevation of UFA results in the membrane resistance to high temperatures, which allows plants to better adjust to the environmental changes. The crucial benefit resulting from the lipids research is that they could serve as the markers of plant physiological status. Moreover, better understanding of the biomembranes remodeling and lipids chemistry allows to generate changes desirable for different sectors of industry like pharmacy or agriculture and food science.
